# Improving Wettability: Deposition of TiO_2_ Nanoparticles on the O_2_ Plasma Activated Polypropylene Membrane

**DOI:** 10.3390/ijms20133309

**Published:** 2019-07-05

**Authors:** Babak Jaleh, Ehsan Sabzi Etivand, Bahareh Feizi Mohazzab, Mahmoud Nasrollahzadeh, Rajender S. Varma

**Affiliations:** 1 Department of Physics, Faculty of Science, University of Bu-Ali Sina, Hamedan 65174, Iran; 2 Department of Chemistry, Faculty of Science, University of Qom, Qom 3716146611, Iran; 3 Regional Centre of Advanced Technologies and Materials, Department of Physical Chemistry, Faculty of Science, Palacky University, Šlechtitelů 27, 783 71 Olomouc, Czech Republic

**Keywords:** PP membrane, O_2_ plasma, TiO_2_ nanoparticles, UV treatment, hydrophilicity

## Abstract

Radio frequency plasma is one of the means to modify the polymer surface namely in the activation of polypropylene membranes (PPM) with O_2_ plasma. Activated membranes were deposited with TiO_2_ nanoparticles by the dip coating method and the bare sample and modified sample (PPM5-TiO_2_) were irradiated by UV lamps for 20–120 min. Characterization techniques such as X-ray diffraction (XRD), Attenuated total reflection technique- Fourier transform infrared spectroscopy (ATR-FTIR), Thermogravimetric analysis (TGA), X-ray photoelectron spectroscopy (XPS), Scanning electron microscope (SEM) and water contact angle (WCA) measurements were applied to study the alteration of ensuing membrane surface properties which shows the nanoparticles on the sample surface including the presence of Ti on PPM. The WCA decreased from 135° (PPM) to 90° (PPM5-TiO_2_) and after UV irradiation, the WCA of PPM5-TiO_2_ diminished from 90° to 40°.

## 1. Introduction

Polypropylene membranes (PPM), due to good porosity, high void volume, and high thermal stability, have wide-ranging applications. The low energy surface and hydrophobicity of the membrane often leads to membrane fouling [1,2]. To address this dilemma, membrane surface treatments have been applied aimed at altering the surface wettability and chemical properties. A wide range of methods have been deployed for altering the surface properties, such as plasma [3,4,5,6,7,8,9], UV irradiation [3], ion irradiation [4] and chemical coating [5,6]. These treatments have been used to attain the following goals: produce special functional groups at the surface for interactions with other functional groups, amend surface energy, change hydrophobicity or hydrophilicity, and alteration of surface morphology [7]. The PPM has been used as a bioinspired substrate for separation applications [8]. Among the present methods for the modification of the surface, plasma treatment is a rather familiar approach [9], which improves the adhesion properties, biocompatibility and wettability [10]. PPM is poor in hydrophobicity and biocompatibility due to the lack of functional groups, which restricts its biomedical usage and possible application in aqueous solution separation and hence the surface modifications induced alterations in bio-compatibility and hydrophilicity. Presently, adsorption and permeation properties of porous membranes can be altered by the deposition of a layer on to their active surface. For example, a hydrophilic layer on the porous membrane can reduce protein binding and enhance flux [2] or alternatively changes can be affected via the addition of nanoparticles such as Al_2_O_3_ [11], ZnO [12], Fe_3_O_4_ [13] and TiO_2_ [14,15,16,17,18,19,20,21]. Among these nanoparticles, TiO_2_ has photo-catalytic and desirable hydrophilicity properties [22]. Studies have been conducted to deposit TiO_2_ nanoparticles on flat polymeric polyethersulfone ultrafiltration (UF) membranes, to decrease the fouling problem [19] with some promising results on the effect of TiO_2_ nanoparticles on UF membranes [23]. Some studies have investigated altering the surface wettability of PPM by means of plasma; compared to the present work, the plasma conditions are different [24,25].

In this work, TiO_2_ nanoparticles were deposited on PP by dip-coating to improve the surface hydrophilicity. At first, the PP membranes were activated by plasma in the range of 1 to 5 min. The high energy species such as electrons, atoms, and radicals in RF plasma interact with the PP surface, leading to the modifications of the surface functionality and the morphology for deposition of TiO_2_. The surface morphology of the PP membrane and PP-TiO_2_ membrane was investigated by SEM, to analyze the distribution of nanoparticles. Moreover, the effect of short UV treatment time on the wettability of the surface of the sample was examined by deployment of characterization techniques to study the ensuing final products.

Wettability has a vital role in the use of polymeric materials in industry and medical science. Oxygen plasma treatment is a common method aimed at fabricated materials in many research fields. Oxygen as a reactant gas that contributes to the fabrication of desirable materials by the surface reaction. During oxygen plasma treatment, the formation of oxygen functional groups ensues. Creating polar groups, oxygen-containing functional groups, has many benefits, especially in changing the wettability of polymers and creating space for bonding nanoparticles [26].

The wetting property plays an important role in the interface of a liquid and a solid surface, especially in polymer applications. External stimuli such as light illumination, temperature, solvents and others, can change surface wetting behavior by changing the morphology of stimuli-sensitive materials. One of the most important ways to change surface wettability is light illumination. There is a variety of responses to light illumination in different materials, but the semiconductor has the same photo-responsive mechanism that has been studied in detail [27]. In brief, since a semiconductor does not have a too large band gap energy, if a photon or light has an energy equal or greater than the band gap energy, the electron in valance band (VB) can absorb energy and jump to the conduction band (CB), resulting in the generation of holes (electron deficiencies) [28]. The charge carriers could recombine by vanishing absorbed energy in the form of light (photon generation) or heat (lattice vibration). Thus, these charge carriers are responsible for carrying out photo-oxidation or photo-reduction reactions [27].

Polymers containing active groups, i.e., oxygen-containing groups (nanoparticles), can be directly absorbed by a chemical reaction between nanoparticles and a polymer surface. In contrast, in inert polymers, nanoparticles are defused and trapped into polymer chains’ free volume [29]. Introducing oxygen-containing groups, e.g., chemical and plasma methods, at the surface of inert polymers contributes to the deposition of nanoparticles [25]. Having a large number of hydrophilic hydroxyl groups, metal oxides show good adherent properties for deposition at the surface of the hydrophilic membrane [30]. It is envisaged that deposition of metal oxides on the membranes with superior chemical stability, such as TiO_2_ or ZrO_2_, would lead to an improvement in their separation performances and to extend their applications in diverse fields.

## 2. Results and Discussion

### 2.1. Scanning Electron Microscope (SEM)

**Figure 1:**
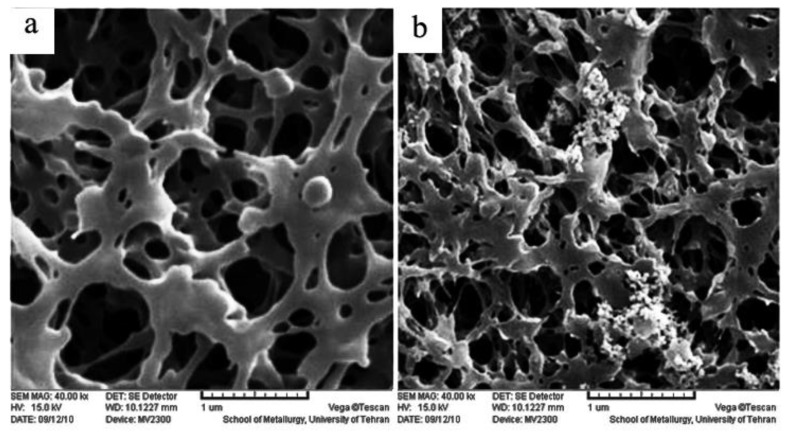
Scanning electron microscope (SEM) images of inactivated PPM (**a**) PPM/TiO_2_ (**b**).

**Figure 2:**
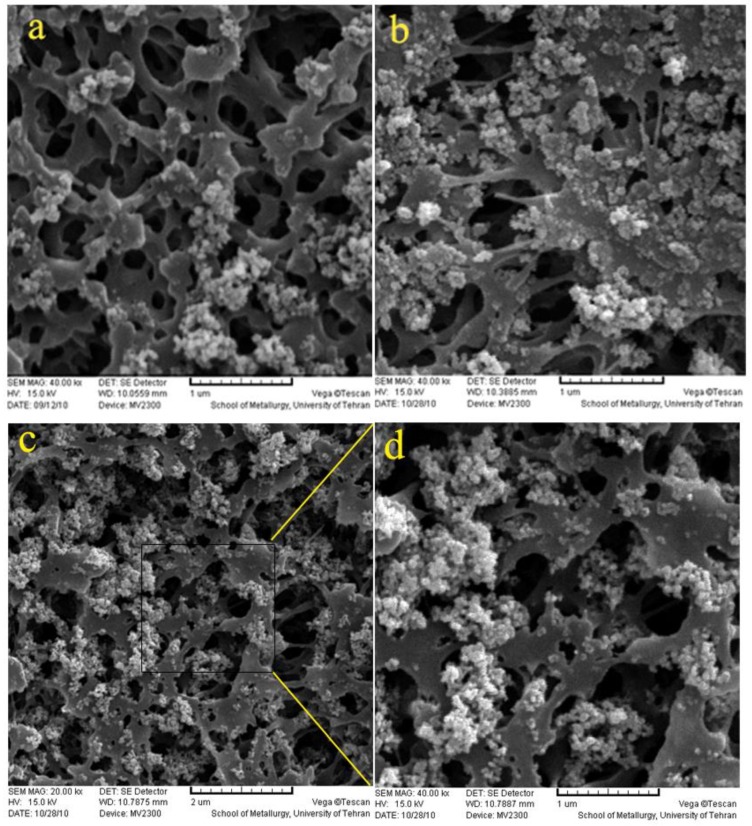
The SEM image of (**a**). PPM1/TiO_2_; (**b**). PPM3/TiO_2_ (**c**,**d**): PPM5/TiO_2_.

To assess the influence of plasma exposure time on the deposition, SEM analysis was performed. The PPM deposited was prepared with different plasma treatment times under the same deposition condition, as mentioned in Section 3.1. The SEM images of pure and deposited PP without plasma treatment are shown in Figure 1 wherein the PP membrane shows a porous surface and lamellar structure, and the deposition of TiO_2_ on the surface of the inactivated PP membrane is not uniform. Besides, the TiO_2_ nanoparticles were aggregated in some regions. The SEM images of S1, S2 and PPM5/TiO_2_ are depicted in Figure 2. As can be observed, the amount of TiO_2_ nanoparticles on the activated samples surface increased by increasing plasma treatment time; comparing deposition degree confirmed that nanoparticles were almost uniformly deposited on the surface of PPM5/TiO_2_. In view of the almost uniform deposition degree, the other analyses were performed for PPM5/TiO_2_ alone.

### 2.2. X-Ray Diffraction (XRD)

**Figure 3:**
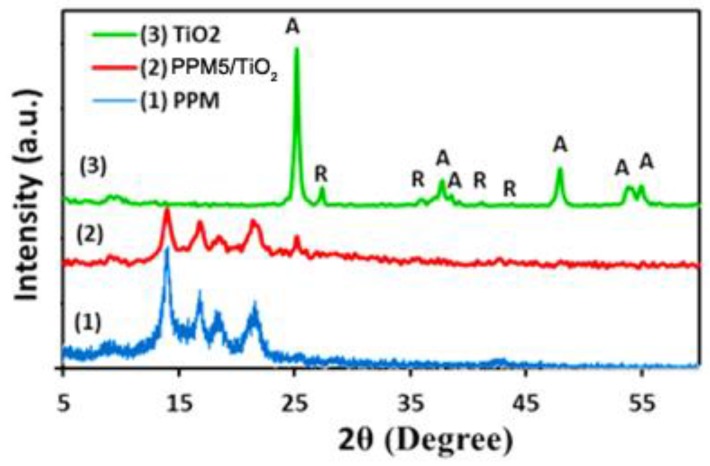
X-ray diffraction (XRD) patterns of (**1**): polypropylene membrane, (**2**): PPM5/TiO_2_ and (**3**): TiO_2_ (A: anatase, R: rutile).

XRD spectra of TiO_2_, PPM membrane and PPM5/TiO_2_ are shown in Figure 3. XRD spectrum of PP shows distinct peaks around 2θ = 14°, 16.9°, 18.5° and 21.8 ° attributed to the crystallographic plans (110), (040), (130), and (041), respectively [31,32]. The pattern of crystalline TiO_2_ nanoparticles shows two characteristic peaks at 2θ = 25.28° and 2θ = 27.4° that indicate the anatase (101) and rutile (110) crystal phases, respectively. The sharp peak positions were in complete agreement with documented reports in the literature [28,33]. The XRD pattern of PP-TiO_2_ shows several peaks for the PP membrane and one peak at 2θ = 25.2° attributed to the presence of TiO_2_ (JCPDS 04-0477). Comparison with the TiO_2_ pattern, the intensity of this peak was weak because of the low amassed value of TiO_2_ on the membrane surface.

### 2.3. Thermal Gravimetric Analysis (TGA)

**Figure 4:**
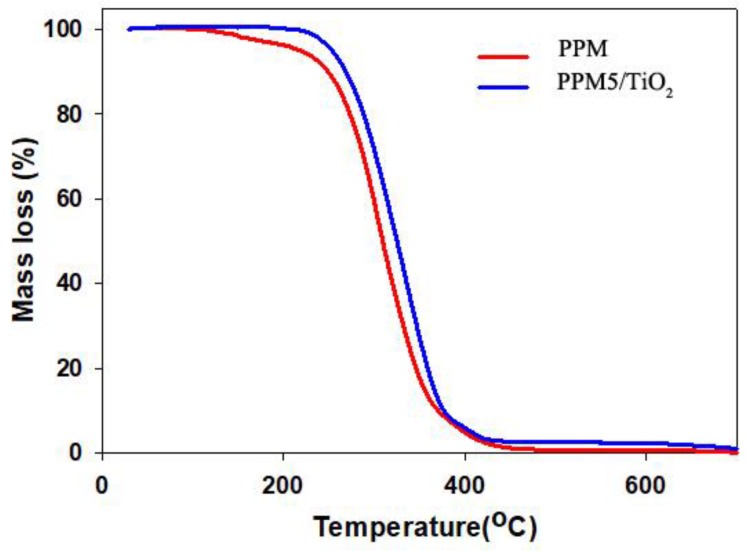
TGA thermograms of PP membrane and PPM5/TiO_2_.

Thermal stability of the PPM and PPM5/TiO_2_. was studied by TGA analysis. Figure 4 illustrates the TGA thermograms of the PP membrane and PP membrane deposited with TiO_2_ nanoparticles. As the curves show, both samples displayed similar behavior with a single mass loss zone. The onset degradation of PP membrane and deposited sample (PPM5/TiO_2_) was observed around 107 °C and 213 °C, respectively. Increasing degradation temperature shows the presence of TiO_2_ nanoparticles had a positive effect on thermal stability. To scrutinize the effect of deposition on thermal stability, three important points are listed in Table 1. According to the results, calculating the differential temperature between the samples during increasing temperature showed a fast decline, probably owing to the splitting of TiO_2_ nanoparticles from the surface of the membrane [34].

### 2.4. Attenuated Total Reflection-Fourier Transform Infrared Spectroscopy (ATR-FTIR)

**Figure 5:**
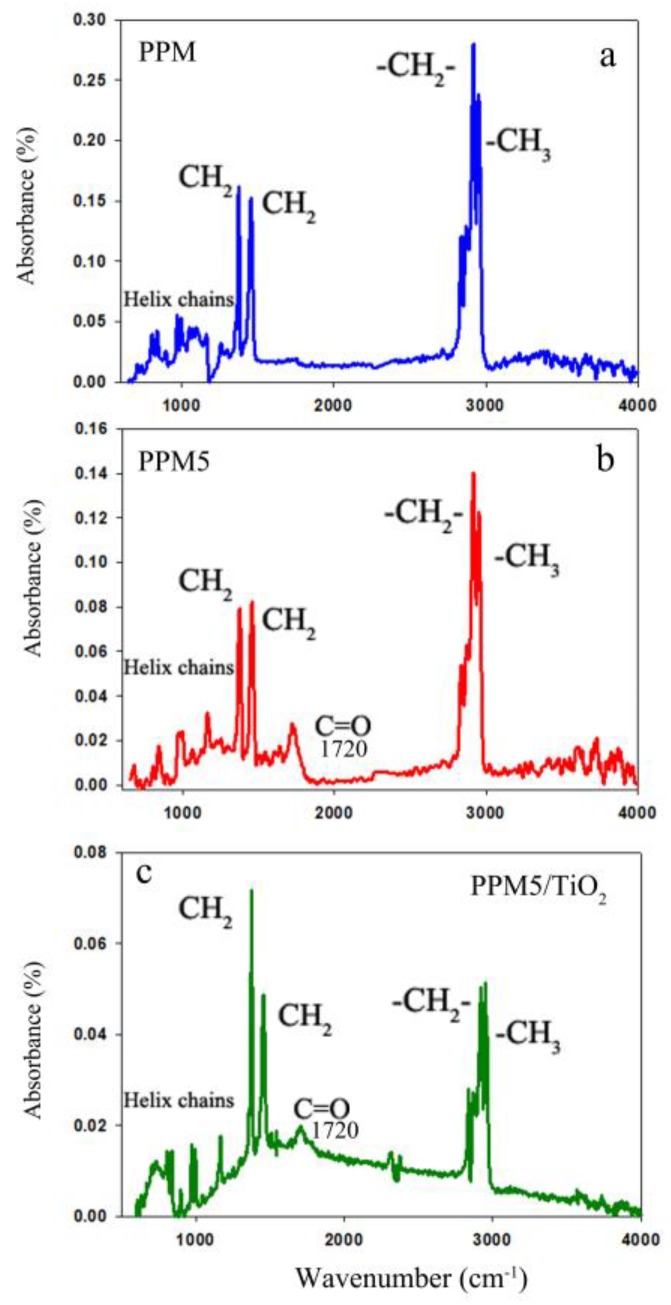
Attenuated total reflection- Fourier transform infrared spectroscopy (ATR-FTIR) spectrum of PPM (**a**), PPM5 (**b**) and PPM5/TiO_2_ (**c**).

To study the influence of plasma and deposition of TiO_2_ nanoparticles on the functional groups of the PP membrane, FTIR-ATR analysis was performed for fresh PPM, activated-PPM and PPM5/TiO_2_. The PP spectrum was considered as a reference, and its peaks are shown in Figure 5a, and they are in good agreement with other reports [32,35,36]. As can be seen in Figure 5b, the peak at 1720 cm ^−1^ (C = O stretching) is a consequence of the O_2_ plasma treatment. The spectra of PPM5/TiO_2_ (Figure 5c) shows an obviously declined intensity of the PP membrane peaks, which may be related to the immobilization of nanoparticles on the surface.

### 2.5. X-Ray Photoelectron Spectroscopy (XPS)

**Figure 6:**
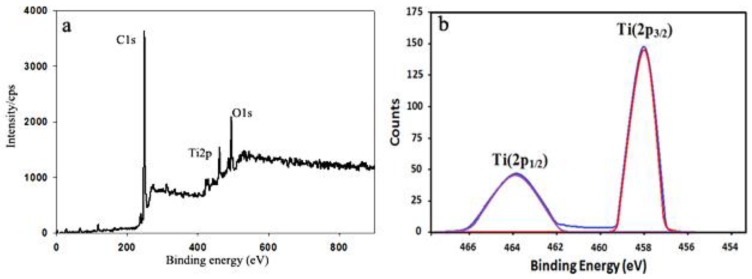
X-ray photoelectron spectroscopy (XPS) spectra of PPM5/TiO_2_ (**a**), Resolved XPS spectra for Ti2p (**b**).

XPS spectroscopy, for the surface characterization of the polymer, was deployed to obtain compelling evidence for the presence of Ti on the surface of the samples. Figure 6 displays the surface chemical changes by precise XPS analysis. In comparison with the membrane [33], two obvious peaks appeared at 458.2 and 531.6 eV corresponding to Ti2p and O1s, respectively. The effect of O_2_ plasma on the polypropylene membrane surface properties was investigated [36]. The XPS results led to the conclusion that oxygen containing functional groups were produced after the plasma treatment. Figure 6b shows the Ti (2p) high-resolution XPS spectrum with two bands in this region. The bands appeared at 463.8 and 458.2 eV binding energies corresponding to the Ti (2p_1/2_) and Ti (2p_3/2_) orbital of Ti atom, respectively.

### 2.6. UV Treatment

In order to investigate the effect of UV treatment on the wettability of PP-TiO_2_, a mercury lamp (TUV 30 W, Philips, Holland) with a wavelength of 254 nm was used; irradiation was performed under ambient conditions and in the range of 20–120 min duration.

When the surface became superhydrophilic, it absorbed more water molecules rather than impurities and organic matter and resisted adsorption. It has been widely reported that TiO_2_ films will turn into superhydrophilic surfaces upon exposure to UV light, the phenomenon being termed photo-induced superhydrophilicity is initiated by the photo-generation of electrons and holes and their migration to the surface [28,37].

### 2.7. Wettability

The sessile drop method was employed to measure the water contact angles (WCAs). The mean value of all WCAs was accomplished by five measurements on the different position of the membrane. To study the influence of irradiation time on wettability, the WCAs were measured as irradiation time. Increasing wettability with a significant change has been reported previously due to the 5 min O_2_ plasma treatment [36]. The increasing hydrophilicity of the activated PP membrane is possibly due to the presence of oxygen-containing functional groups, which has been corroborated by XPS results.

**Figure 7:**
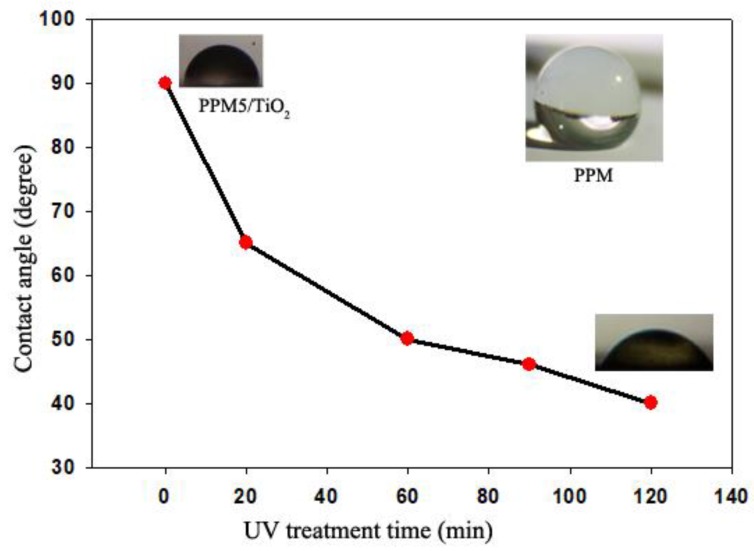
WCA (water contact angles) as a function of UV treatment time.

The increasing wettability (hydrophilic functional groups) of the PP membrane contributes to a good deposition of TiO_2_ nanoparticles on the surface of the sample. The WCA measurements for PPM activated by O_2_ plasma (5 min) and deposited with TiO_2_ at different UV irradiation times are shown in Figure 7; WCAs decreased from 135° to 90° after plasma irradiation and 90° to 40° after UV irradiation. It has been widely reported that TiO_2_ will turn into hydrophilic surfaces when exposed to UV light [37]. Compared to other reports [24], the plasma’s step in the present work was more effective in changing the wettability of PPM.

When the TiO_2_ surface was irradiated with UV light, an electron-hole pair was generated. The reaction could take place between the electron-hole pairs and absorbed H_2_O and O_2_ molecules. This mechanism led to an increase in wettability on the surface of TiO_2_.

## 3. Materials and Methods

PP membranes with a diameter of 47 mm, a thickness of 190 µm and pore sizes of 0.22 µm were used for O_2_ plasma treatment by radio frequency plasma (EMITECH KX1050, East Sussex, UK) TiO_2_ powder (Degussa, Berlin, Germany, P-25) was used in these studies.

### 3.1. Preparation of Samples

Initially, to remove any chemical contamination from the PP membrane, it was washed with acetone, then dried at room temperature. Subsequently, the modification was carried out by means of 25 W O_2_ plasma at 0.1 mbar pressure at different times, 1, 3 and 5 min. The TiO_2_ suspensions were prepared using appropriate concentrations (0.05 wt%) of TiO_2_ in pure ethanol (Merck, Kenilworth, NJ, USA). The TiO_2_ suspension was homogenized via ultrasonication (40 °C for 20 min) and the suspension was deposited on the activated membrane by means of the dip-coating method three times with a 2 mm·s ^−1^ speed. The samples were dried between each deposition cycle. The specimens were named as PPMx/TiO_2_, where x represents the plasma treatment time (x = 1, 3 and 5). Then the membranes were washed with distilled water, dried and finally illuminated by UV lamp (TUV 30 W, Philips, Amsterdam, Netherlands) for 20–120 min; the distance of being 20 cm from the lamp.

### 3.2. Membrane Characterization

X-ray diffraction (XRD, Unisantis xmd300, Georgesmarienhutte, Germany) measurements were recorded in 2θ in the range of 5–60°. The surface morphologies of PP and PPM5 deposited TiO_2_ nanoparticles (PPM5/TiO_2_) were examined by SEM (Philips, model XL30). Change of functional groups and influence nanoparticles deposition on the surface were studied using FT-IR-ATR (Bruker Alpha, Yokohama, Japan). Thermal gravimetric analysis (TGA) was accomplished by using a heating rate of 10 °C/min from 30 °C to 800 °C under air flow radiation. X-ray photoelectron spectroscopy (XPS) was recorded via an Al Kα X-ray source at 1486.6 eV. Finally, the alteration in wettability was examined using the WCA measuring system.

## 4. Conclusions

In continuation of our ongoing studies on PP membrane activated by means of O_2_ plasma treatment, the present work dwells on the wettability as influenced by the deposition of TiO_2_ nanoparticles on activated samples and UV irradiation. To overcome the hydrophobic properties of PPM, O_2_ plasma treatment was utilized for various durations. Consequently, oxygen-containing functional groups appeared on the surface of PPM which facilitated the deposition of TiO_2_ nanoparticles by the dip-coating method. The SEM depicts that most parts of TiO_2_ were attached to the membrane surface as clusters. The XPS and XRD analyses confirmed the presence of TiO_2_ nanoparticles at the surface of PPM. FTIR analysis affirmed the successful formation of oxygen-containing groups on the PP surface after a very short O_2_ plasma treatment. The water contact angle (WCA) results led to the conclusion that significant improvement of the hydrophilicity of PPM5/TiO_2_ was associated with the deposition of TiO_2_. Deployment of UV treatment led to better effects in improving the hydrophilicity. Similarly, the flux and work efficiency of the membrane could be improved because of the enhanced hydrophilicity.

## Figures and Tables

**Table 1:** Mass-loss temperature obtained from thermogravimetric analysis (TGA) thermogram of PPM and PPM5/TiO_2_.

Samples	Mass Loss Temperature (±2 °C)		
	T_O_	T_50_	T_90_
PP membrane	107	310	380
PPM5/TiO_2_	213	358	394

The T_O_, T_50_ and T_90_ performed temperature of onset degradation, 50% and 90% mass-loss, respectively.
